# High-flow nasal cannula: recommendations for daily practice in pediatrics

**DOI:** 10.1186/s13613-014-0029-5

**Published:** 2014-09-30

**Authors:** Christophe Milési, Mathilde Boubal, Aurélien Jacquot, Julien Baleine, Sabine Durand, Marti Pons Odena, Gilles Cambonie

**Affiliations:** Département de Pédiatrie Néonatale et Réanimations, Pôle Universitaire Enfant, CHRU de Montpellier, Montpellier, 34000 France; Unidad de Cuidados Intensivos Pediatricos, Hospital Universitario Sant Joan de Deu, Universitat de Barcelona, Esplugues de Llobregat, Barcelona, 08950 Spain; Réanimation Pédiatrique, Hôpital Arnaud de Villeneuve, 371 avenue du doyen G. Giraud, 34295 Montpellier CEDEX 5, France

**Keywords:** PICU, High-flow nasal cannula, Bronchiolitis, Asthma

## Abstract

High-flow nasal cannula (HFNC) is a relatively new device for respiratory support. In pediatrics, HFNC use continues to increase as the system is easily set up and is well tolerated by patients. The use of nasal cannula adapted to the infant’s nares size to deliver heated and humidified gas at high flow rates has been associated with improvements in washout of nasopharyngeal dead space, lung mucociliary clearance, and oxygen delivery compared with other oxygen delivery systems. HFNC may also create positive pharyngeal pressure to reduce the work of breathing, which positions the device midway between classical oxygen delivery systems, like the high-concentration face mask and continuous positive airway pressure (CPAP) generators. Currently, most of the studies in the pediatric literature suggest the benefits of HFNC therapy only for moderately severe acute viral bronchiolitis. But, the experience with this device in neonatology and adult intensive care may broaden the pediatric indications to include weaning from invasive ventilation and acute asthma. As for any form of respiratory support, HFNC initiation in patients requires close monitoring, whether it be for pre- or inter-hospital transport or in the emergency department or the pediatric intensive care unit.

## Review

### Introduction

Over the last decade, high-flow nasal cannula (HFNC) has increasingly been used for oxygen delivery in neonatology departments, gradually replacing nasal continuous positive airway pressure (CPAP). Its use in pediatrics departments is more recent and generally is restricted to children with moderate bronchiolitis. The cannula was first employed in intensive care units (ICUs), then in emergency departments, and today is finding use during pre- or inter-hospital transport.

Clinicians are quite rightly raising questions about where it should be positioned among the systems of noninvasive respiratory support, such as high-concentration face masks and nasal CPAP. Its mode of action is original and complex. Initiating HFNC is relatively simple, but close monitoring is essential. Since the critical review of HFNC use in ill infants, children, and adults [1], additional physiological and clinical data have been reported, particularly in infants with acute viral bronchiolitis. The range of indications for HFNC is also likely to broaden in the future, and further studies are therefore needed to ensure that the guidelines for use are evidence-based.

### Mechanism of action

HFNC is designed to administer a heated and humidified mixture of air and oxygen at a flow higher than the patient’s inspiratory flow [1]. There is currently no single, simple definition of high flow. In infants, it usually refers to the delivery of oxygen or an oxygen/room air blend at flow rates greater than 2 L/min [2]. Some authors adjust the flow rates on body weight and recommend using 2 L/kg/min, which provides a degree of distending pressure [3-5] and reduces the work of breathing [6]. In children, flow rates >6 L/min are generally considered high flow [1]. High flow presents several advantages over conventional ‘low-flow’ oxygen therapy in terms of humidification, oxygenation, gas exchange, and breathing pattern.

#### Gas mixture conditioning

HFNC provides a relative humidity of nearly 100% with the gas warmed to between 34°C and 37°C. Compared with ‘low-flow oxygenation’ or the high-concentration oxygen mask, HFNC improves patient tolerance by reducing the sensation of respiratory distress and mouth dryness [7]. Moreover, Hasani et al. observed tracer movements and demonstrated improved mucociliary clearance [8]. In comparisons of HFNC and conventional oxygen therapy, this effect is thought to explain the drop in exacerbation episodes and the improved quality of life in adult patients with chronic obstructive pulmonary disease (COPD) [9].

Another benefit of gas conditioning is the improved inspiratory flow, which further increases the feeling of comfort. Heated and humidified gas diminishes the resistance in the nasal mucosa induced by dry and cold gas [10], a point that should not be neglected given that these resistances make up nearly 50% of the total resistance of the respiratory system.

#### High flow

Several studies have shown that a flow higher than the patient’s inspiratory flow provides better oxygen delivery than low-flow oxygen therapy or the high-concentration oxygenation mask. This observation has been explained as the effect of a high flow on the oropharyngeal dead space, with the idea being that the high flow of oxygen ‘washes out’ the end-expiratory oxygen-depleted gas. In the next breath, the patient inhales pure oxygen [7,11,12]. Dead space washout also reduces CO_2_ rebreathing.

The extrathoracic dead space is proportionally two to three times greater in children than in adults. It may measure up to 3 mL/kg in newborns and becomes similar to the adult volume only after 6 years of age (0.8 mL/kg) [13]. Consequently, the younger a child is, the greater the effect of a high flow on oxygenation and CO_2_ clearance [14].

#### Generated pressures

A high-flow mixture is likely to create a maximum positive pharyngeal pressure of about 6 cm H_2_O during expiration [3,15-17]. The pressure is determined not only by the flow, but also by the ratio of the prong/nostril fit and whether or not the mouth is closed. The inter- and intra-individual variations are nevertheless quite wide [18].

**Figure 1:**
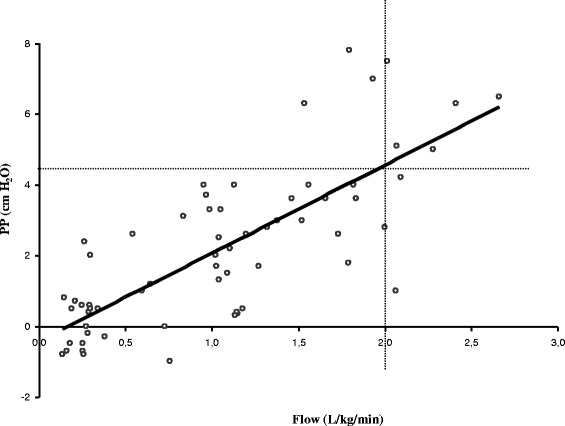
**Pharyngeal pressure (PP) over the course of a gradual increase in flow.** The flow is indexed to patient weight (*R* = 0.77, *p* < 0.001). A flow >2 L/kg/min is associated with mean pharyngeal pressure >4 cm H_2_O (sensitivity 67%, specificity 96%, positive predictive value 75%, negative predictive value 94.5%). Adapted from Milési et al. [3].

**Figure 2:**
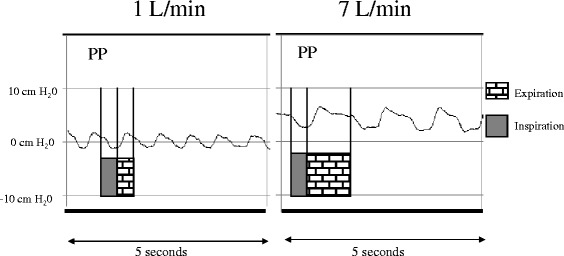
**Recording of the pharyngeal pressure (PP) at 1 and 7 L/min in an infant.**

In a physiological study of infants with acute viral bronchiolitis, we measured pharyngeal pressure over the course of a gradual increase in flow up to 7 L/min (Figure 1) [3]. When we indexed the flow to patient weight, we observed that the average pressure with a flow of 2 L/kg/min was about 4 cm H_2_O. Unfortunately, despite the overall shape of the curve, we could not predict whether a higher flow would provide greater pressure. The pharyngeal pressure at a rate of 1 L/min appeared like a sine wave around the air pressure, being negative during inspiration and positive during expiration (Figure 2). The sinusoidal shape persisted when we increased the flow, but the two pressure components (inspiratory and expiratory) became positive after 7 L/min, thereby generating real CPAP.

The pressures generated by the device prevent pharyngeal collapse, which may be very pronounced in some diseases. It reduces obstructive apnea and supports the inspiratory effort when patient flow is limited. In infants with bronchiolitis, Pham et al. recently showed that HFNC reduced the electrical activity of the diaphragm and decreased esophageal pressure swings, confirming the effectiveness of this therapy to reduce the work of breathing [6]. The effects of CPAP differ with the ventilation phase. Positive pressure at the beginning of inspiration may compensate the inspiratory burden related to auto-positive end-expiratory pressure (auto-PEEP) and facilitate inspiratory flow. Positive pressure during expiration prevents small airway collapse (stenting effect), increases the expiratory time and reduces the auto-PEEP.

The favorable effect of this technique on the ventilation/perfusion ratio has not been clearly established. This suggests the need for caution when HFNC is used in the management of respiratory failure type 1. In this case, the ventilation/perfusion mismatch dominates the pathophysiology, whereas alveolar ventilation is relatively preserved [19].

#### Reduced energy expenditure

The burden on the respiratory muscles may be very high in children with obstructive respiratory distress. The high energy expenditure may lead to respiratory muscle failure and recourse to mechanical ventilation. The risk of decompensation is particularly high in young infants because their respiratory muscles are poorly equipped with oxidative fibers, which increases muscle vulnerability to excessive and prolonged work.

Several features of HFNC suggest positive effects on energy expenditure compared with conventional oxygen therapy, notably preserved mucociliary function, prevention of atelectasis, and decreased inspiratory work [3,6,8-10,14-20].

### Side effects and monitoring

HFNC stands out from conventional oxygen therapy because it provides a heated and humidified air flow that counteracts the unpleasant sensation of a dry mouth [7]. This nuisance is one of the major sources of discomfort cited by ICU patients. Compared with other systems delivering CPAP, cutaneous tolerance is better with fewer skin lesions reported [21]. However, like any respiratory support system, this device has drawbacks. For example, the noise level reaches about 80 dB. The decibel level is correlated with the flow and may be higher than that generated by other CPAP systems [22].

Recently, three episodes of pneumothorax and pneumodiastinum were reported during HFNC use [23]. The risk of air leak syndrome could be associated with an inappropriate prong size that occludes the nostril lumen [24]. Another difficulty with this device as a substitute for CPAP is the great intra- and inter-patient variation in the pressures generated in the airways [18]. Flow rates may be titrated to the evolving status of respiratory distress, but the safety of this practice is uncertain because subsequent changes in generated pressure are not measured.

Finally, the greatest risk in using HFNC, as for any noninvasive ventilation (NIV) strategy, is that recourse to more invasive management may be delayed in cases of respiratory decompensation. Some authors have thus suggested that the failure of NIV, because it delays the recourse to mechanical ventilation, may actually increase mortality/morbidity. Up to now, this observation was been confined to the adult population [25]. In children, the risk of HFNC failure, defined as intubation requirement, ranges from 8% to 19% [15,26-29] and reaches nearly 30% when escalation in respiratory support is also taken into consideration [4]. In children younger than 2 years, HFNC failure may occur within 7 to 14 h [28,29], whereas with other NIV strategies, failure was usually observed in the first 2 h following initiation [30]. In the absence of randomized controlled trials, it is impossible to determine whether this difference is due to the characteristics of the population, the variability in disease progression, or the respiratory support itself. HFNC should therefore be initiated in an emergency department or a pediatric ICU that has sufficient staff to closely monitor the patient’s clinical course and that is well trained to recognize the early signs of failure. After several hours of stability, the infant may be transferred to a conventional ward, depending on hospital policy.

### HFNC initiation in practice (Figure **3**)

**Figure 3:**
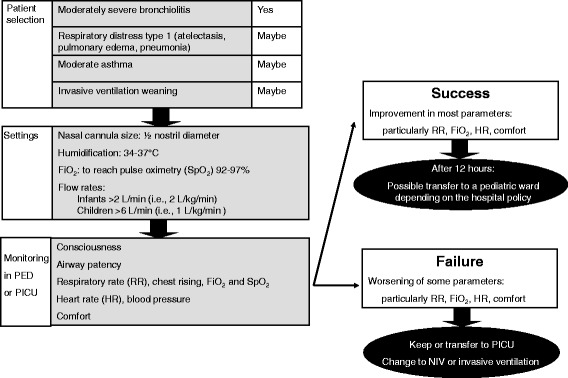
**HFNC initiation and monitoring.** PED, pediatric emergency department; PICU, pediatric intensive care unit; RR, respiratory rate; HR, heart rate; NIV, noninvasive ventilation.

The HFNC system has few parts: the cannula, a flow generator, an air/oxygen blender, and a respiratory gas humidifier.

#### Where to initiate HFNC

Although most studies of HFNC therapy have focused on ICUs, recent works have shown that HFNC can be used to manage moderate respiratory distress in emergency departments [29] and during pre- or inter-hospital transport [31]. One of the advantages of HFNC is that it requires minimal technical skill to set up and apply. Nevertheless, initiating this type of respiratory support requires advanced experience in managing acute pediatric respiratory illness, adequate technical monitoring and a high staff/patient ratio. The risk of decompensation requires very close monitoring in a setting that is equipped for rapid implementation of invasive ventilatory support. Discharge from the ICU and transfer to a pediatric ward can be considered only once the continued improvement of these children is well underway. The ward admitting the child will nevertheless need to provide close surveillance and be equipped with a centralized alarm system for early detection of respiratory failure or signs of decompensation.

#### Cannula

The prong caliber is adapted to the nostril size in order to allow for leakage and avoid overpressure phenomena. The prong diameter should be about half that of the nostril [24]. It may be useful for infants to reduce mouth leaks with a pacifier.

#### Generator

Three types of gas generators are currently available:

- The first type uses an air/oxygen blender and is connected to a system to humidify and heat the gas. Several devices are available: Optiflow System® (Fisher and Paekel, Auckland, New Zealand), Precision Flow® (Vapotherm, Exeter, UK), and Comfort-Flo® (Teleflex Medical, Durham, NC, USA). There may be a pressure relief valve that cuts off the flow when a predetermined pressure in the circuit is reached. The practical consequence of this valve is flow limitation depending on the cannula size.
- The second type uses a turbine + humidifier (Airvo2®, Fisher and Paekel, Auckland, New Zealand). This system has the advantage of not requiring an external source of gas, except oxygen. This device cannot be used with neonates and its start-up is sometimes a bit long compared with other types.
- The third type is based on a CPAP or conventional ventilator with an HFNC breathing circuit connected to the humidifier.

#### Settings

In infants, flow rates are greater than 2 L/min [2] and may be adjusted to body weight, i.e., 2 L/kg/min [3-6]. In children, flow rates are greater than 6 L/min [1] and may be up to 20 to 30 L/min [15,32], thus closer to 1 L/kg/min. FiO_2_ is set to achieve target saturation between 92% and 97%. The gas temperature is set around 37°C in order to reach optimal humidification [33,34]. If the patient’s room is cool, it may be useful to insulate the tubing or to use breathing circuits with heating wires to limit condensation and the spray of water droplets into the child’s nostrils. If the phenomenon continues, the heater temperature can be reduced to a minimum of 34°C.

### The indications for HFNC

Despite the advantages of this technique, the quality of the literature dealing with a pediatric population remains poor. The Cochrane Library deemed that no study was able to provide indications and guidelines for HFNC therapy in pediatric patients with a high level of evidence [2]. Similar conclusions were expressed about the use of HFNC in the specific situation of infants with acute viral bronchiolitis [35]. In 2014, recommendations are still based on extrapolations from observational or physiological studies, but not on evidence. For clinical practice, HFNC seems feasible in most of the populations currently managed with NIV, and sometimes, it appears to be better tolerated.

The most prudent course would be to restrict HFNC therapy to mild forms of respiratory distress and situations of discomfort or interface intolerance. Whatever the etiology of the respiratory distress, observational studies suggest significant success rates [15,26,27,36-38]. However, HFNC use in about 490 children with respiratory distress (bronchiolitis, pneumonia or asthma) was associated with NIV failure and recourse to mechanical ventilation in 8% of the cases [29]. Unsurprisingly, the failures were observed in the most severely ill patients who presented with significant respiratory acidosis and remained tachypneic after initiation.

#### Acute viral bronchiolitis

HFNC has most often been evaluated in populations with acute viral bronchiolitis, with several studies comparing the efficacy and tolerance of HFNC with different CPAP systems [4,26,35,39].

Clinically, these infants show signs of severe obstructive lung disease, with a marked increase in respiratory resistance and reduced dynamic compliance. The ‘trapping’ phenomenon is exacerbated by the change in ventilatory pattern, being characterized by rises in the respiratory rate and in the ratio of inspiratory time (Ti) over the total respiratory cycle time (Ti/Ttot ratio) [40]. The gradual increase in end-expiratory volume generates a positive end-expiratory pressure or auto-PEEP. The work of breathing is increased because, at each inspiration, patients need to use their muscles to offset the auto-PEEP and then continue the work for generating an inspiratory flow despite the increased airways resistance.

Measurement of esophageal pressure helps to quantify the inspiratory effort required to ensure alveolar ventilation in this situation. The effort is about six times higher in infants with severe bronchiolitis than that observed in healthy infants [40]. Applying oropharyngeal pressure equivalent to the auto-PEEP generates an inspiratory flow as soon as the inspiratory muscles begin working and thus reduces the inspiratory burden [3,6,40,41]. In addition, CPAP may keep small airways open by enlarging the diameter (‘stenting’ effect), which in turn would reduce respiratory system resistance.

Several ‘before-after’ observational studies have suggested the interest of HFNC on both physiological [3,6] and clinical grounds [5,28,36-39], including a decreased rate of intubation as compared with historical controls prior to HFNC [26,27]. From this perspective, a failure rate comparable to that of CPAP performed with a nasopharyngeal tube was reported [4], while a recent randomized control study reported efficiency comparable to hypertonic saline [42]. However, no study to date has provided a direct demonstration of the risk of mechanical ventilation requirement as most of the patients included in these studies were not affected by severe forms of bronchiolitis. Therefore, it seems reasonable to reserve NIV/CPAP for severe bronchiolitis and to limit HFNC use to moderate forms of the disease.

#### Withdrawal of invasive ventilation

In the neonatal population, weaning from invasive ventilation is one of the main indications for HFNC, with recent randomized studies demonstrating efficiency comparable to that of CPAP [43,44]. In the adult population, as well, a few studies have suggested the advantages of using HFNC for this indication, but the number of patients is still modest [45]. These results need to be confirmed in larger populations [46]. In infants younger than 18 months, a recent randomized controlled trial compared HFNC to conventional oxygen therapy in the 48-h post-extubation after cardiac surgery [47]. HFNC had no influence on PaCO_2_ values, which was the primary objective. However, its use appeared safe and improved PaO_2_ in these patients. This pioneering work, along with the positive experience reported in this area with newborn and adult patients, should encourage studies on HFNC use for the withdrawal of invasive ventilation in infants and children. For the moment, application of HFNC in this context is based only on the clinical judgment of the practitioner and initiated with great caution.

#### Asthma

From a physiological point of view, HFNC for asthmatic patients seems attractive. As in bronchiolitis, CPAP may reduce the burden on the inspiratory muscles related to auto-PEEP. Use of heated and humidified gas also limits the bronchoconstriction induced by cold dry gas. Theoretically, the high gas flow should improve the distribution of inhaled treatments. However, this effect remains a subject of controversy, as the dose of bronchodilator received varies from 0.5% to 25% of the administered dose [48,49]. Distal bronchodilator delivery might be improved by positioning the aerosol upstream of the humidifier, choosing an ultrasonic nebulizer over a pneumatic nebulizer or even using heliox gas as the vector [50,51]. However, the literature is scant on the use of high flow in this indication. Kelly et al. described the largest observational study to date, which included 38 children under 2 years of age presenting with a severe asthmatic attack [29]. Experience with HFNC for this indication is particularly lacking and this must be emphasized. For instance, in our PICU, we limit HFNC use to the mildest asthmatic attacks. Use of another type of NIV becomes mandatory if tachypnea and/or signs of respiratory distress do not improve within 1 h of HFNC initiation.

## Conclusions

HFNC use is increasing in pediatric wards, despite the lack of clearly established benefits in the medical literature. The indication most cited in the publications is moderately severe bronchiolitis in infants, but recent reports suggest HFNC may also be effectively and safely applied to a broader spectrum of patient ages and diagnoses [29,37,38]. The system is very attractive because of its simplicity and excellent tolerance. On a practical level, this treatment should be initiated in the emergency department or the pediatric ICU in order to evaluate its effectiveness and identify as early as possible the signs of failure requiring a more appropriate respiratory support system.
